# Prevalence of Antibiotic-Resistant Bacteria and Antibiotic-Resistant Genes and the Quantification of Antibiotics in Drinking Water Treatment Plants of Malaysia: Protocol for a Cross-sectional Study

**DOI:** 10.2196/37663

**Published:** 2022-11-21

**Authors:** Zuraifah Asrah Mohamad, Sophia Karen Bakon, Mohd Azerulazree Jamilan Jamilan, Norhafizan Daud, Lena Ciric, Norazah Ahmad, Nor Asiah Muhamad

**Affiliations:** 1 Health Risk Assessment Unit, Environmental Health Research Centre Institute For Medical Research, National Institutes of Health Ministry of Health of Malaysia Shah Alam Malaysia; 2 Nutrition, Metabolic, and Cardiovascular Research Centre Institute For Medical Research, National Institutes of Health Ministry of Health of Malaysia Shah Alam Malaysia; 3 Engineering Services Division Ministry of Health of Malaysia Putrajaya Malaysia; 4 Department of Civil, Environmental and Geomatic Engineering University College London London United Kingdom; 5 Infectious Disease Research Centre Institute For Medical Research, National Institutes of Health Ministry of Health of Malaysia Shah Alam Malaysia; 6 Sector for Evidence-Based Healthcare National Institutes of Health Ministry of Health of Malaysia Shah Alam Malaysia

**Keywords:** drinking water, river, safe, antibiotic, resistant, antimicrobial, sanitation, Malaysia, Asia, bacteria

## Abstract

**Background:**

Antimicrobial resistance is a known global public health threat. In addition, it brings serious economic consequences to agriculture. Antibiotic resistance in humans, animals, and environment is interconnected, as proposed in the tricycle surveillance by the World Health Organization. In Malaysia, research and surveillance of antimicrobial resistance are mainly performed in clinical samples, agricultural settings, and surface waters, but no surveillance of the drinking water systems has been performed yet. Hence, this policy-driven study is a combined effort of microbiologists and engineers to provide baseline data on the magnitude of antimicrobial resistance in the drinking water systems of Malaysia.

**Objective:**

The aim of this study was to study the baseline level of antibiotic-resistant bacteria in the drinking water distribution systems of Malaysia by collecting samples from the pretreatment and posttreatment outlets of water treatment plants in a selected state of Malaysia. We aimed to determine the prevalence of antibiotic-resistant bacteria, the occurrence of antibiotic-resistant genes, and the level of antibiotics present in the drinking water systems.

**Methods:**

This is a laboratory-based, cross-sectional study in a selected state of Malaysia. Water samples from 6 drinking water treatment plants were collected. Samples were collected at 3 sampling points, that is, the intake sampling station, service reservoir outlet station, and the distribution system sampling station. These were tested against 7 types of antibiotics in triplicates. Samples were screened for antibiotic-resistant bacteria and antibiotic-resistant genes and quantified for the level of antibiotics present in the drinking water treatment plants.

**Results:**

We will show the descriptive statistics of the number of bacterial colonies harvested from water samples grown on Reasoner’s 2A agar with or without antibiotics, the occurrence of antibiotic-resistant genes, and the level of antibiotics detected in the water samples. The sampling frame was scheduled to start from November 2021 and continue until December 2022. Data analysis is expected to be completed by early 2023, and the results are expected to be published in mid-2023.

**Conclusions:**

This study provides baseline information on the status of the antimicrobial-resistant bacteria, the presence of resistance genes as contaminants, and the level of antibiotics present in the drinking water systems of Malaysia, with the aim of demonstrating to policymakers the need to consider antimicrobial resistance as a parameter in drinking water surveillance.

**International Registered Report Identifier (IRRID):**

DERR1-10.2196/37663

## Introduction

### Background

**Figure 1:**
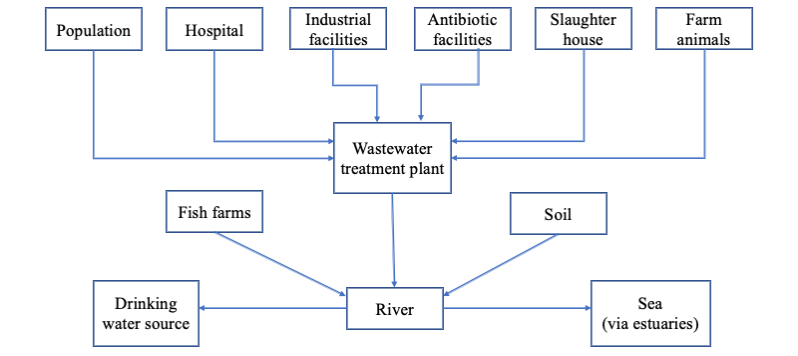
Route of antibiotic-resistant bacteria and antibiotic-resistant genes from various anthropogenic sources to a drinking water source. Adapted from Stalder et al [2].

Access to clean and safe water is of the utmost importance worldwide. For general well-being, human beings use water for daily consumption and hygiene purposes. Water also serves as an important factor for maintaining a sustainable environment and plays an important role in climate change issues. Unfortunately, environmental water also plays a prominent role in the spread of antibiotic-resistant genes (ARGs) and antibiotic-resistant bacteria (ARB). Over 80% of all wastewater in low-income countries is estimated to be discharged untreated directly into rivers, lakes, or the oceans [1]. High-risk areas such as waste discharges of pharmaceutical production facilities, hospitals, and other health care facilities have been identified as specific hotspots for ARGs and ARB. Antibiotics released into the environment may also promote the selection of ARB and ARGs, which find their way into the soil and natural water bodies [2] (Figure 1).

Antimicrobial resistance (AMR) occurs when microorganisms develop the ability to resist antimicrobial treatment designed to inhibit their growth and kill them. Bacteria, as the most extensively studied for AMR in both clinical and environmental settings, can exchange resistance genes between species in a microbial population [3]. Infected humans and animals can act as reservoirs and spread these resistant bacteria into the environment through various routes. Furthermore, misuse of antimicrobial agents can accelerate the process of AMR.

Tracking of the spread of antibiotic-resistant microorganisms provides insights into the transmission of antibiotic-resistant strains and resistance genes from humans or animals to the environmental reservoir and back to human use and consumption [4,5]. Studies have suggested that aquatic systems such as drinking water sources [6], wastewater effluents [7], and wastewater discharge from hospitals act as reservoirs for antibiotic-resistant microorganisms [8]. Metagenomics (the study of genetic material from environmental samples) analysis has revealed that wastewater treatment plants are a hotspot for ARGs and their mobile genetic elements [9,10]. The nutrient-rich wastewater brings together microbes from the environment, humans, and animals originating from domestic, industrial, agricultural, and medical activities, and allows the spread of ARGs between microbial species [11]. Although wastewater treatment plants can reduce the concentrations of pathogens, ARB and their ARGs have been detected in effluents and biosolids [12] and effluent-receiving rivers [7]. The effluent-receiving river, which eventually is used as a drinking water source, can also contain resistant bacteria from fish farms and soil.

The World Health Organization calls for coordinated actions to minimize the emergence and spread of AMR by providing technical assistance to countries to develop national health action plans and urging more research and development on AMR. As of October 2021, 148 countries have finalized their National Action Plan, which aligns with the objectives of the Global Action Plan on Antimicrobial Resistance [13]. In Malaysia, the collaborative effort between the Ministry of Health and the Ministry of Agriculture and Agro-based Industry has resulted in a Malaysian Action Plan on Antimicrobial Resistance (MyAP-AMR) 2017-2021 [14]. The MyAP-AMR details out plans to minimize AMR in both health care practices and agricultural sectors; their aims among others are to increase the awareness of antimicrobials, promote appropriate usage of antibiotics in clinical settings, and decrease pollution influenced by the direct use of antibiotics in poultry farms [14]. Furthermore, contamination of drinking water sources, including water pollutants such as antimicrobials/antiseptics, is ranked third in the list of “Top 10 Environmental Health Issues in Malaysia” released by the National Environmental Health Action Plan, Malaysia [15]. The United Nations 2030 Agenda for Sustainable Development has listed “Goal 6: Clean water and sanitation” as one of its 17 sustainable development goals to stimulate actions between 2016 and 2030 in areas of critical importance [1].

A few published studies in Malaysia have reported the presence of pharmaceutical residues from surface water in Malaysia. The presence of human pharmaceutical products, which include antidiabetic agents, antihypertensive agents, hypolipidemic agents, β-2 adrenergic receptor agonists, antihistamines, analgesics, and sex hormones, in river water and sewage effluent were reported, but no antibiotics were detected [16]. Al-Qaim et al [17] reported the presence of caffeine, prazosin, enalapril, carbamazepine, simvastatin, hydrochlorothiazide, diclofenac sodium, and mefenamic acid in surface waters. Praveena et al [18] reported the presence of pharmaceutical residues, including antibiotics, in 3 rivers in Selangor, Malaysia. These researchers found that ciprofloxacin was detected in all the samples, with the highest concentration in the rivers [18]. In another study, Lee et al [19] evidenced carbapenem-resistant *Vibrio parahaemolyticus* isolated from marine and freshwater fish samples in Selangor, which suggested the spread of AMR into the food chain [19]. Presumptive *Escherichia coli* isolates that showed resistance to chloramphenicol, penicillin, tetracycline. and kanamycin were detected from soil at recreational parks in villages in Sabah, Malaysia [20]. Antibiotic-resistant *E. coli* and genes (*tet* and *sul*) were also detected in wastewater effluents and the river waters of Larut River, Perak, which were associated with anthropogenic activities [21]. Ho et al [22] reported that 86% of *Enterococcus faecalis* from River Melayu in Johor State, Malaysia, was multidrug-resistant and associated with a local sewage treatment plant and other anthropogenic activities [22].

### Monitoring of AMR in the Drinking Water System

Drinking water treatments have previously been shown to act as a source of antibiotic resistance, and water distribution systems could serve as an important reservoir for microbial resistance [23]. Although ARB have been discovered in tap water around the world, little is known about their specific patterns of resistance [24]. Chen et al [6] reported the distribution of *E. coli* from 2 drinking water sources in Hangzhou city, China, which were resistant to tetracycline, ampicillin, piperacillin, trimethoprim, sulfamethoxazole, and chloramphenicol. [6]. Gu et al [25] reported 317 ARG subtypes in drinking water treatment plant (DWTP) bulk water and sand biofilm with widespread detection of genes encoding bacitracin, multiple drugs, and sulfonamide in Guangzhou, China. In Germany, enterobacterial *ampC* resistance genes were detected from wastewater, surface water, and drinking water biofilms [26]. In another study by Xi et al [27], ARGs (*cat*, *cmr*, *bla*_TEM_, *blaSHV*, *suII*, *suIII*) and heterotrophic ARB (total heterotrophic ARB) were detected in all finished water and tap water samples from several cities in Michigan and Ohio, United States. The levels of bacteria in the source water were higher than those in tap water; however, the levels of ARB in tap water were higher than those in finished water, demonstrating that there was regrowth of bacteria in the drinking water distribution systems. Furthermore, ARGs were found at higher concentrations in tap water than in finished water and source water [27]. ARG *int1*, conferring resistance to ampicillin, was detected in coliform isolates from restaurants in Bangladesh [28], and *blaNDM-1* was isolated from drinking tap water in Karachi, Pakistan [29]. In South Africa, the total count of coliforms in random household tap water was reported to be higher than that in raw water intake during winter and summer [30].

The presence of biofilm-producing bacteria such as *Pseudomonas* spp has been associated with microbial resistance in the drinking water system. Bacterial biofilm is formed by a consortium of bacteria and acts as a mechanism for better survival to make its producers more resistant to antibiotics and disinfectant chemicals compared to planktonic cells [31]. Biofilm detachment upon treatment with a disinfectant in drinking water distribution systems leads to an increasing amount of ARB in tap water [32]. A study in Bulgaria found significant differences in the ARB population in biofilms from 4 DWTPs [33]. In the United Kingdom, significant correlations were reported between surviving bacteria from chlorinated drinking systems and resistance (measured by the minimum inhibitory concentrations) against tetracycline, sulfamethoxazole, and amoxicillin [34].

Outbreaks in hospitals caused by multidrug-resistant *Pseudomonas aeruginosa* have been linked to the formation of biofilms in wastewater systems [35] and tap water [36] contaminations as well as in domestic drinking water plumbing systems [37]. In South China, Su et al [38] reported the presence of bacteria and *tet* genes (tetracycline resistance) in tap water after treatment although it was much lower than that in source water and suggested that *Pseudomonas* spp played a role in the proliferation and dissemination of ARGs [38]. In France, multiresistant *Pseudomonas* spp (but not *E. coli*) was detected in treated (treatment: flocculation, deposition, and sand filtration, and chlorination reservoir) drinking water from both spring water and the tap system [39]. Bergeron et al [40] reported the presence of ARGs, *tetA* and *sul1*, and ARB, including *E. coli*, *Enterobacter cloacae*, *P. aeruginosa*, and *Klebsiella pneumoniae*, in the raw intake water in Louisiana, United States. No ARGs or ARB were found in the treated and distributed water, although bacterial DNA in the form of 16s rRNA was consistently found [40].

Although there is no conclusive evidence on the “safe limit” of ARB or ARGs in drinking water worldwide, several studies have been conducted in other countries, including China, which have indicated the presence of antibiotic residues, ARB, and ARGs in their water systems. These data have been used to warrant further research and put up new efforts to improve their DWTP systems (eg, to improve the filtration system). For example, Liu et al [41] reported that hybrid carbon membranes of thick graphene oxide and activated carbon effectively remove tetracycline from water by 98.9% [41]. Su et al [38] reported that ARGs still existed in tap water after treatment and the use of granular activated carbon filtration in the DWTPs in China increased ARG abundance [38].

Several hospital-acquired outbreaks caused by gram-negative bacteria such as *Acinetobacter baumannii* and *P. aeruginosa* have been associated with contaminated water distribution systems [35,42,43]. The presence of *E. coli* has been used as an indicator of fecal contamination in drinking water. Multidrug-resistant *E. coli* has been isolated from food sources [44], urinary isolates [45], as well as hospital and municipal effluents [46]. However, it is more interesting to elucidate whether there is a relationship between environmental-driven resistance and clinically acquired resistance in bacteria or whether the resistance is strictly either environmental or clinical.

AMR is an important global issue that needs to be addressed holistically so as to prevent people from dying owing to ineffective antibiotic treatment and to avoid antibiotic resistance eventually, leading us to the postantibiotic era [47]. In Malaysia, research and surveillance of AMR are mainly performed on clinical samples, agricultural settings, and surface water (rivers) but none on the drinking water system. Hence, this may increase the risk of exposure to ARB and ARGs, which are not being monitored at present.

The aim of this study is to address the abovementioned gaps by determining the prevalence of ARB and the occurrence of resistant genes (ARGs) in a natural water source, drinking water treatment system, and water distribution outlets in Malaysia. We will do this by investigating the most common microbial species and their phylogenetic relationships before the water treatment and after the water treatment and in different DWTPs by determining the occurrence of ARB and ARGs and by quantifying the level of antibiotics.

## Methods

### Study Design

**Figure 2:**
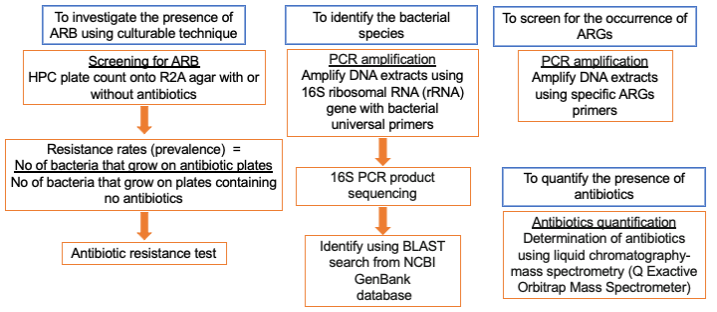
Flow of the experimental work. AMR: antimicrobial resistance; ARB: antibiotic-resistant bacteria; ARG: antibiotic-resistant gene; BLAST: Basic Local Alignment Search Tool; HPC: heterotrophic plate count; NCBI: National Center for Biotechnology Information; PCR: polymerase chain reaction; R2A: Reasoner's 2 agar.

This is a cross-sectional study using laboratory-based methodologies (Figure 2). The DWTPs in the Malaysian state of Selangor will be chosen because of several factors such as the availability of published evidence of AMR in surface water and freshwater fish, large population, presence of both industrial and agricultural settings, and logistic factors from the sampling location to the laboratories.

**Figure 3:**
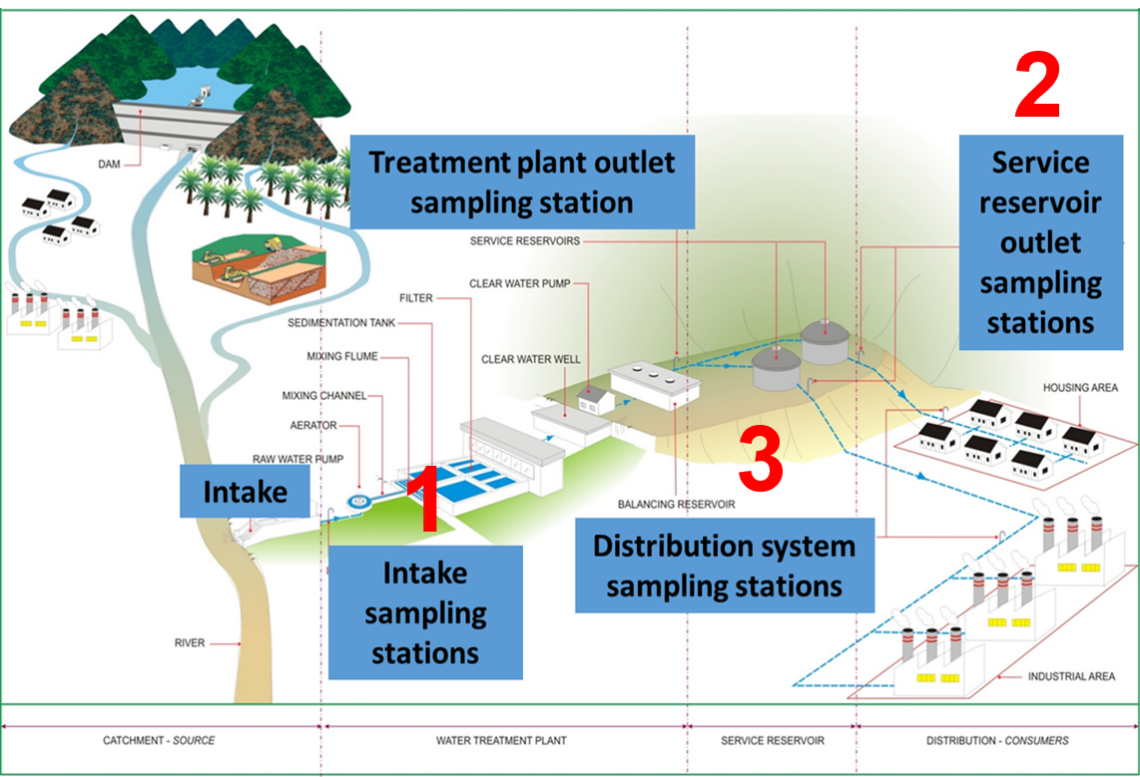
Schematic diagram of a drinking water treatment plant in Malaysia (Source: Engineering Services Division, Ministry of Health, Malaysia).

We will use simple random sampling to choose a sample. We will randomly generate a number for each DWTP by using Excel and identify the sample. We assume that all the DWTPs are homogenous (Figure 3). The sample size will be chosen based on the manageable samples for processing. Samples will be collected at 3 sampling points, that is, intake sampling station, service reservoir outlet station, and distribution system sampling station for 7 antibiotics (amoxicillin, chloramphenicol, ciprofloxacin, gentamicin, tetracycline, sulfamethoxazole, and vancomycin), and the analysis will be performed in triplicates. In this preliminary stage, antibiotics are selected based on the most commonly prescribed antibiotics in Malaysia [48] and surveillance data from the Antibiotic Resistance Surveillance Reference Laboratory, Institute for Medical Research, Malaysia [49]. Therefore, the number of samples to be collected from each DWTP was calculated as follows: 3 sampling points × 7 antibiotics × 3 replicates = 63 samples. Sampling will be performed at 3 points as follows: point 1, intake sampling point (river source, immediately before going into DWTPs); point 2, service reservoir outlet sampling station (posttreatment DWTP); and point 3, distribution system sampling station/main tap (immediately before distribution to households).

### Sample Size and Power Calculation

Sampling size was calculated using prevalence [50], as follows:

Number of DWTPs in Selangor = 34

Prevalence range of heterotrophic ARB in a drinking water plant [27]: 1.17%-39.55%


n = z^2^
*P* (1-p)/d^2^

Assuming the prevalence (p) would be 40%, p=0.40; *d*=0.05


= 1.96^2^ (0.3955) (0.6045)/0.05^2^

= 3.8416 (0.239)/0.0025

= 0.9181/0.0025

= 367.24

Where n=sample size, z=1.96 for a confidence level (α) of 95%, p=assumed prevalence, and d=precision (corresponding to effect size).

### Inclusion and Exclusion Criteria

All DWTPs are monitored under the National Drinking Water Quality Program, Selangor State Health Department, Ministry of Health of Malaysia. There are no exclusion criteria.

### Microbiological Analysis

#### Water Sampling

Samples will be handled properly by trained laboratory personnel to ensure aseptic techniques, and other procedures will be performed according to the quality management system for microbiological analysis. Water sampling will be performed following the Standard Methods for the Examination of Water and Wastewater [51]. Briefly, samples for microbiological analysis will be collected in 1-L sterile wide-mouth screw-capped bottles. To maintain the sample’s integrity, a proper sample collection technique will be applied to ensure that water samples will be representative of the water being tested. Sampling at raw water intake locations will not be performed if there is rain or any unusual reported cases of contamination at the DWTPs. Samples will be collected from 3 points, namely, raw water intake sampling point, posttreatment outlet tap at the DWTP, and distribution tap at the residential area. Grab sampling will be performed with no manipulation of volume, such as pouring or adding to the sample to avoid contamination. To avoid exterior contamination at the posttreatment and main tap, the tap will be sterilized thoroughly using a flame torch. Sampling points at the distribution system will be chosen to demonstrate water quality throughout the network and to ensure that no localized contamination occurs. Water will be allowed to run through the tap for up to 5 minutes prior to sample collection to clear the piping line, which may harbor bacteria that do not reflect the actual water quality. Samples will be kept in an ice-packed cooler maintained at 4 °C and transported back to the laboratories for immediate processing within 48 hours.

#### Screening for Antibiotic-Resistant Heterotrophic Bacteria

We will be using culture-dependent methods and molecular techniques to determine the prevalence of ARB, identify them, and investigate the occurrence of ARGs present in DWTPs to answer each specific objective. A heterotrophic plate count on Reasoner’s 2A agar (Oxoid) will be used to determine the ARB in the collected water samples. Bacteria in river water (sources) are expected to be present at much higher concentrations at approximately 350 colony-forming units (CFUs)/100 mL [52]. Samples from river water sources that have high concentrations of bacteria will be serially diluted to obtain single bacterial colonies for further analysis. The membrane filtration method will be used for testing lower bacterial concentrations from posttreatment and main tap samples. A previous study estimated that the number of bacteria that can be harvested from chlorinated drinking water is approximately up to 200 CFUs/100 mL [34]. Negative control plates and an aseptic technique will be applied to ensure that there is no contamination occurring during laboratory processing. Plates will be incubated at 37 °C for 2 days followed by 27 °C for another 5 days according to Gao et al [53] with some modifications. For river water samples, plates will be incubated at 37 °C for 24 hours where optimum bacterial colony size is achieved. The experiment will be performed in triplicates, instead of duplicate plates for each sample. The Kirby-Bauer test for antibiotic susceptibility (also called the disc diffusion test) will be used to determine the susceptibility of bacterial isolates to various antibiotics, namely, amoxicillin, ciprofloxacin, vancomycin, gentamycin, chloramphenicol, sulfamethoxazole, and tetracycline (Oxoid).

#### Screening for ARGs

The polymerase chain reaction method will be used for the rapid and accurate identification of bacteria by using universal primers targeting the 16S rRNA gene [54]. The 16S rRNA gene sequencing is a highly useful tool for identifying bacteria at the genus/species level and in differentiating between closely related bacteria species [55]. The DNA sequences obtained will be subjected to phylogenetic analysis. Phylogenetic network analysis is used to elucidate any occurrences of horizontal gene transfer of 16S rRNA in bacteria [56]. The development of phylogenetic trees is an important step for determining the diversity of resistant bacteria in the environment and for characterizing emerging pathogens (if there are any) to provide information to the relevant stakeholders. Cell-associated ARGs in the drinking water system (from isolated resistant bacteria) and cell-free ARGs from water samples (which may add potential risk) in DWTPs will be investigated. Eleven specific primers specific to genes of interest will be used to amplify the genes and to screen for the presence of each ARG in each water sample (Table 1). Genomic DNA will be extracted using a commercial DNA isolation kit according to the manufacturer's instructions. The DNA extracts will be kept at –20 °C until further analysis.

**Table 1:** List of the published primers representing the occurrence of bacterial DNA and antibiotic-resistant genes [40,57,58].

Gene name	Resistance mechanism
16S rRNA gene	N/A^a^
*ermB*	Ribosomal protection
*Sul1*	Enzymatic modification
*tetA*	Efflux
*tetW*	Ribosomal protection
*tetX*	Enzymatic modification
*mecA*	β-lactam binding protein
*BlaNDM-1*	Hydrolysis
*BlaOXA-23*	Enzymatic degradation
*BlaOXA-51*	Efflux
*BlaTEM*	Hydrolysis
*VanA*	Amino acid cleavage

^a^N/A: not applicable.

### Chemical Analysis

Samples for antibiotic quantification (Figure 3) will be collected in 1-L high-density polyethylene sample bottles. High-performance liquid chromatography will be used to quantify the level of the 7 selected antibiotics. A high-performance liquid chromatography system (Textbox 1 and Table 2) will be used to quantify the level of antibiotics from all samples following the method by Kim et al [59].

Before quantification, samples will be preconcentrated and eluted to achieve satisfactory accuracies and sensitivities [59]. The procedure/steps will be carried out as follows:

1. Sample collection and treatment
2. Sample preconcentration using a nitrogen evaporator
3. Sample clean-up by automated solid-phase extraction
4. Analysis using liquid chromatography–mass spectrometry (Q Exactive Orbitrap Mass Spectrometer, Thermo Fisher Scientific)

**The high-performance liquid chromatography system used in this study.**

**Internal standard**

1. Amoxicillin-d4 (Toronto Research Chemicals)

**External standard**

1. Amoxicillin trihydrate (Dr Ehrenstorfer GmbH)
2. Ciprofloxacin hydrochloride (Dr Ehrenstorfer GmbH)
3. Vancomycin hydrochloride (Dr Ehrenstorfer GmbH)
4. Gentamycin sulfate (Dr Ehrenstorfer GmbH)
5. (+/-) chloramphenicol (Cambridge Isotope Labs Inc)
6. Sulfamethoxazole (Dr Ehrenstorfer GmbH)
7. Tetracycline hydrochloride (Dr Ehrenstorfer GmbH)

**Analytical column**

Waters Xbridge C18 Column 50 mm×2.1 mm id, 2.5 µm at 35 °C

Solvent gradient

A: Water liquid chromatography-mass spectrometry grade (Merck)

B: Acetonitrile with 0.1% (v/v) formic acid (Sigma-Aldrich)

Flow: 0.8 mL/min

**Table 2:** Solvent gradient for the determination of antibiotics by high-performance liquid chromatography.

Retention time (min)	Flow rate (mL/min)	Mobile phase A (water liquid chromatography-mass spectrometry grade) (%)	Mobile phase B (acetonitrile with 0.1% (v/v) formic acid) (%)
0	0.80	95	5
1	0.80	95	5
12	0.80	70	30
13	0.80	0	100
17	0.80	0	100
17.1	0.80	95	5
23	0.80	95	5

### Ethical Considerations

No clinical samples will be collected. This study has been exempted from ethics approval by the Medical Research and Ethics Committee, Malaysia. Data will be disseminated to the Drinking Water Quality Surveillance Program (DWQSP), Engineering Services Division, Ministry of Health, as our main stakeholders and collaborator, and through a peer-reviewed publication or presentation following approval from the Ministry of Health, Malaysia.

### Statistical Analysis

A paired 2-sided *t* test will be used to compare the prevalence of heterotrophic ARB in source water and tap water. Data in replicates for the quantification of antibiotics (in parts per trillion) will be recorded in Excel. All DNA sequences obtained will be analyzed using the Chromas program (Informer Technologies Inc) for any missing nucleotides or errors. The Basic Local Alignment Search Tool will be used to determine the species identities of the sequences and to find the pattern of occurrence between sampling points and between DWTPs. The sequences will be aligned, and then a phylogenetic relationship will be constructed using the Lasergene software (DNASTAR).

### Data Management

Data will be recorded in Microsoft Excel. DNA sequences will be kept in FASTA files. All positive ARB samples will be recorded for resistance profiles and will be kept as environmentally derived ARB culture collections for future studies.

## Results

### Outcomes

This project was funded in June 2020. The sampling frame was scheduled to start from November 2021 and continue until December 2022. To date (July 2022), we have sampled 4 DWTPs at 3 points, that is, the river (source), posttreatment outlet (DWTP), and the main distribution tap. Results will be reported as the prevalence of ARB in the drinking water system and the level of antibiotics present in the drinking water system a**s** the primary outcome. As the secondary outcome, bacteria isolated from the primary outcome will be tested to investigate whether they are carrying ARGs in the drinking water system. Descriptive statistics will be performed on the number of bacterial colonies harvested from water samples grown on Reasoner’s 2A agar with or without antibiotics, the level of antibiotics detected in the water samples, and the occurrence of ARGs. Data analyses are ongoing and expected to be published in 2023.

### Microbiological Analyses

The prevalence of heterotrophic ARB in source water will be compared with that in other sampling points. Only strains that are able to grow on Reasoner’s 2A agar containing antibiotics will be subjected to analysis for antibiotic susceptibility and ARGs. Each antibiotic-resistant strain will be inoculated on Muller-Hinton media (Oxoid) for testing. Results from the antibiotic susceptibility test (Kirby-Bauer disk assay) will be compared to Clinical and Laboratory Standards Institute disk measurement standards for specific bacteria and antibiotics. However, for isolates that are not included in the Clinical and Laboratory Standards Institute list, results will be interpreted by adopting the European Committee on Antimicrobial Susceptibility Testing database or by considering inhibition diameters ≥10 mm as susceptible [60]. The zone of inhibition will be measured to determine the susceptibility or resistance of the organism to each drug and will be reported accordingly as susceptible, intermediate, and resistant. The presence of the 16S rRNA amplicon indicates bacterial DNA, and specific gene amplicons indicate the presence of ARGs. Data will be recorded as follows: the total heterotrophic plate count in the water samples will be reported as CFUs/100 mL. The resistance rate (prevalence) and multiresistant index will be reported in percentages. The percentage of resistance rate (prevalence) and multiresistance index will be calculated as follows:

Resistance rates (prevalence%) = Number of bacteria that grow on antibiotic plates/number of bacteria that grow on plates containing no antibiotics

Multiresistance index = Number of antibiotics to which isolate is resistant/number of antimicrobials to be tested

The presence of ARGs will be marked by determining the amplification by the specific primers of each gene of interest. DNA will be visualized using 2% agarose gel. Positive bands of sample DNA using 16S rRNA primer sets indicate the presence of bacterial DNA, while positive bands in samples using specific ARG primer sets show the presence of ARGs at the particular sampling points.

### Chemical Analyses

The preconcentration technique will be used for each antibiotic to achieve parts per trillion detection. The concentrations of the antibiotics will be quantified and determined whether the levels correspond with the ARB at each site.

## Discussion

### Principal Findings

From our preliminary analysis in 3 DWTPs, we found multidrug-resistant *Enterobacteriaceae* as well as heterotrophic ARB in river water sources. The spread of ARB has been associated with wastewater discharge [61] and other anthropogenic activities [21]. Of the 56 isolates of *E. coli* and *Salmonella* spp tested, 24 (40%) were multidrug-resistant isolates, 14 (58%) were resistant to at least 3 antibiotics, 4 (17%) were resistant to 4 antibiotics, 5 (21%) were resistant to 5 antibiotics, and 1 (4%) was resistant to 6 antibiotics. Similarly, the presence of multidrug-resistant *E. coli* in rivers [62,63] and drinking water sources has been reported in other countries such as China [6], United States [27], and Iran [64], thereby highlighting the importance of efficient effluent treatments from AMR hotspots before being discharged into the rivers [7].

The outcomes of this study would provide a basis for holistic research in AMR by determining the extent of AMR present in Malaysia and the likely paths of transmission from the environment to the public. The prevalence data on antibiotic resistance generated from this study will also help the relevant stakeholders in Malaysia to steer evidence-based policies to control and prevent the possible transmission of ARB in drinking water systems to humans [65] and to complement with other current AMR research and monitoring in Malaysia.

The DWTP in Malaysia consists of a river catchment as the water source, water treatment plant, service reservoir, and finally, the distribution outlet from where it goes to every house (Figure 3). The monitoring of the drinking water system in Malaysia currently falls under the DWQSP, the Ministry of Health. To date, Malaysia is still lacking in the research and monitoring of antimicrobials (ARB and ARGs) in drinking water systems (source water, DWTP, and tap water). The current microbiological parameters being monitored are fecal coliforms and *E. coli*. In the future, to have this parameter stated in the National Water Quality Standard would help to indicate whether the water is safe enough for daily consumption. Furthermore, we would be able to inform other ministries to be aware of the proper disposal of leftover antibiotics, which can end up in wastewater or rivers, and inform them about the importance of efficient drinking water treatment for human consumption.

We have identified several strengths in this study; first, this study provides baseline data on ARB in the drinking water system of Malaysia. Second, this study will illustrate the burden of ARGs in the drinking water treatment systems in urban areas. Third, these results will be used to provide evidence and increase the precision of the quantitative estimates of exposure related to ARGs in drinking water and finally provide baseline data for AMR in national safe drinking water. We anticipate limitations such as the recovery of bacteria from low nutrient media when cultivated in enriched media, which will require optimization of the incubation period and temperature, and a lack of reference data on minimum inhibitory concentrations to interpret all types of bacteria, especially the less commonly known bacteria present in a water system.

There are several impacts anticipated from this study. First, we may elucidate the magnitude of the problem by providing baseline information on the level of ARB and ARGs present in our drinking water system in Selangor, Malaysia. Second, we may improve the water treatment system to be more sustainable, safe, and clean, particularly for long-term daily water consumption [66]. Countries like China have incorporated intervention studies by testing various filtration systems to continuously improve their water treatment system. Finally, this study may improve the public health system and environmental health.

Findings from this study will be used to determine whether AMR is a problem in our drinking water by providing information on the status of ARB and the presence of ARGs in drinking water systems. Depending on the magnitude of AMR in the drinking water system, further actions/key measures can be taken (future research or by providing evidence to the stakeholders) to reduce or eliminate the contaminants from entering our drinking water system. This study would help to draw a holistic picture of the occurrence of antibiotic resistance in the environment, apart from other anthropogenic sources such as human effluents that may contribute to antibiotic resistance, by considering water as a potential reservoir for humans to be exposed to antibiotic resistance. This study will provide information on the status of ARB and the presence of resistance genes as contaminants in drinking water systems, which are not being monitored in the National Drinking Water Quality program by the Ministry of Health at present. Further actions and key measures can be taken for future research or expert advice to reduce or eliminate contaminants from entering our drinking water system. This study will broaden a research niche in AMR concerning the environment and human health risks in Malaysia.

### Conclusion

AMR is a public health challenge that requires the concerted efforts of multiple agencies. AMR management in clinical and agricultural settings is more established compared to that in DWTPs in Malaysia. The output from this study will provide evidence and key measures to benefit and assist the current DWQSP to reduce the risk of antibiotic resistance transmission to the public. Rationally, if the resistance is decreased at the local level, it can help prevent the global antibiotic resistance crisis from growing even bigger.

### Recommendation

Data from this study will be used to embark on future research based on the tricycle (human, food and environment) approach by incorporating the possible sources of pollutants. This will enable us to determine the prevalence of AMR within the same localities and thus enable researchers to elucidate the possible route of transmission of AMR in a defined setting, especially into the rivers as drinking water sources.

## Abbreviations

AMR: antimicrobial resistance
ARB: antibiotic-resistant bacteria
ARG: antibiotic-resistant gene
CFU: colony-forming unit
DWTP: drinking water treatment plant
DWQSP: drinking water quality surveillance program
MyAP-AMR: Malaysian Action Plan on Antimicrobial Resistance

## Data Availability

Data will be kept in the Ministry of Health and will be disseminated following approval from the Director-General of Health, Malaysia.
